# School sessions are correlated with seasonal outbreaks of medically attended respiratory infections: electronic health record time series analysis, Wisconsin 2004–2011

**DOI:** 10.1017/S0950268818003424

**Published:** 2019-03-08

**Authors:** J. L. Temte, J. G. Meiman, R. E. Gangnon

**Affiliations:** 1 Department of Family Medicine and Community Health, University of Wisconsin, Madison, Wisconsin, USA; 2 Department of Population Health Sciences and Biostatistics & Medical Informatics, University of Wisconsin, Madison, Wisconsin, USA

**Keywords:** Influenza, respiratory infections, transmission

## Abstract

Increased social contact within school settings is thought to be an important factor in seasonal outbreaks of acute respiratory infection (ARI). To better understand the degree of impact, we analysed electronic health records and compared risks of respiratory infections within communities while schools were in session and out-of-session. A time series analysis of weekly respiratory infection diagnoses from 28 family medicine clinics in Wisconsin showed that people under the age of 65 experienced an increased risk of ARI when schools were in session. For children aged 5–17 years, the risk ratio for the first week of a school session was 1.12 (95% confidence interval (CI) 0.93–1.34), the second week of a session was 1.39 (95% CI 1.15–1.68) and more than 2 weeks into a session was 1.43 (95% CI 1.20–1.71). Less significant increased risk ratios were also observed in young children (0–4 years) and adults (18–64 years). These results were obtained after modelling for baseline seasonal variations in disease prevalence and controlling for short-term changes in ambient temperature and relative humidity. Understanding the mechanisms of seasonality make it easier to predict outbreaks and launch timely public health interventions.

## Introduction

Acute respiratory infection (ARI) seasonality is generally believed to stem from three main factors: the cyclical nature of host immunity, weather variations and changes in host behaviour, most notably in how people interact with each other [1, 2]. Close person-to-person contact, particularly among school-age children, is frequently cited as an important example of the latter. An association between school sessions and ARI outbreaks has long been observed [3], and many researchers have proposed school attendance as an important driver of early autumn and winter spikes in ARIs. Yet research demonstrating this effect is relatively limited. Measles was one of the first viruses that showed a strong correlation with school sessions [2, 4, 5]. More recent research on influenza [6, 7] has also suggested a link, and rhinovirus has demonstrated autumnal peaks that correlate closely with the onset of school [8]. Any attempt to discern a correlation between school attendance and seasonality, however, is necessarily complicated by other potential drivers that these studies do not address.

Of all potential correlates of seasonality, temperature and relative humidity are the most widely studied. Colder temperatures are associated with increases in ARIs, and studies have largely confirmed an inverse relationship in temperate regions. Inverse relationships between viral prevalence and temperature have been shown for common viral pathogens, including influenza, respiratory syncytial virus (RSV), rhinovirus, adenovirus, coronavirus and human metapneumovirus [9–13]. Relative humidity has been studied extensively, albeit with less consistent findings. Several studies suggest that higher relative humidity correlates with increased prevalence of RSV, rhinovirus, adenovirus and coronavirus [9–11, 14–16]. Research on the role of temperature and relative humidity in bacterial causes of seasonal ARI is more limited as seasonal ARI outbreaks are primarily viral in children [17, 18] and often viral in adults [19, 20].

Understanding ARI seasonality informs public health interventions aimed at limiting the spread of respiratory infection. Thus, we designed this study to determine the level to which school attendance contributes to seasonal outbreaks of all-cause respiratory infections, as assessed within a primary care electronic health record (EHR) database. To account for confounding meteorological factors, we included temperature and relative humidity in the model of seasonality.

## Methods

### Respiratory infection cases

Weekly ARI case counts were estimated using a composite of ‘all-cause’ respiratory infection diagnoses derived from the University of Wisconsin, Department of Family Medicine and Community Health's clinical data warehouse (CDW). The CDW aggregates EPIC Systems EHR data that is extracted daily from a network of 28 ambulatory practices, primarily in the south-central portion of the state. The records for approximately 800 000 ambulatory visits are available annually and represent approximately 2.5% of Wisconsin's total population. Weekly ARI diagnoses from May 2004 to July 2011 were determined by ICD9 codes as shown in Table 1.

**Table 1.** All cause acute respiratory infections – ICD9 Coding

*381.0–382.9 Otitis media*
(381) Nonsuppurative otitis media and eustachian tube disorders
(382) Suppurative and unspecified otitis media
*460.00–466.99 Acute respiratory infections*
(460) Acute nasopharyngitis (common cold)
(461) Acute sinusitis
(462) Pharyngitis, acute
(463) Tonsillitis, acute
(464) Acute laryngitis and tracheitis
(465) Acute upper respiratory infections of multiple or unspecified sites
(466) Acute bronchitis and bronchiolitis
*480–488.1 Pneumonia and Influenza and H1N1*
(480) Viral pneumonia
(481) Pneumococcal pneumonia
(483) Other bacterial pneumonia
(484) Pneumonia in infectious diseases not classified elsewhere
(485) Bronchopneumonia, organism unspecified
(486) Pneumonia, organism unspecified
(487) Influenza
(488) Influenza due to identified avian influenza virus

**Fig. 1.**
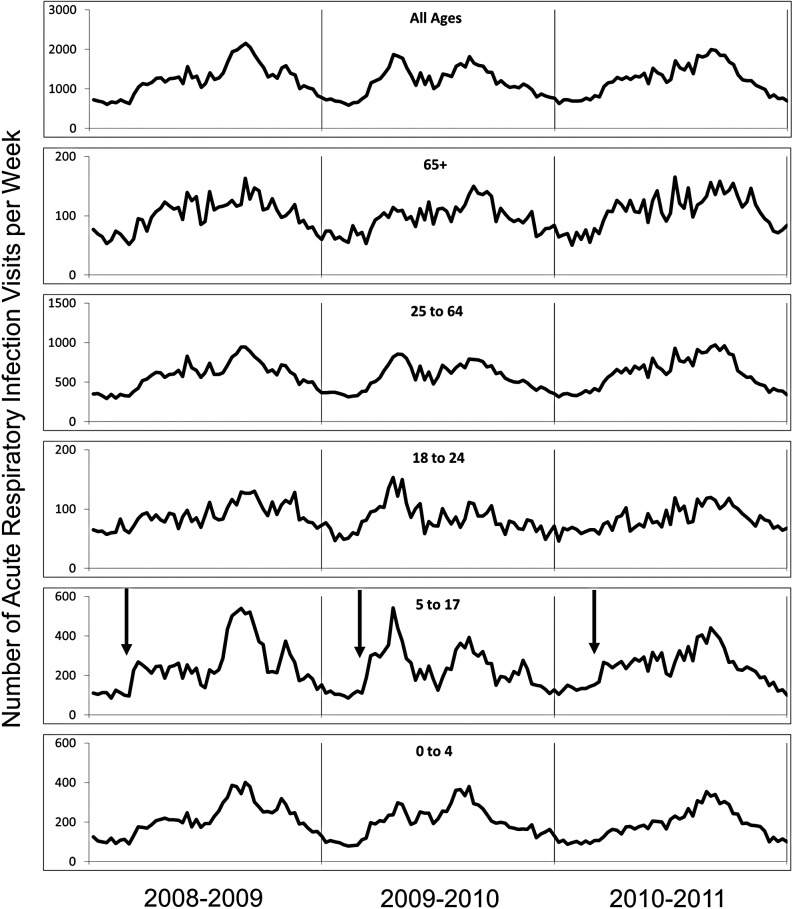
ARI counts by age group (July 2008 through June 2011). Arrows in panel for children, ages 5–17 years, indicates start of the academic year.

The local database was originally developed for ongoing assessments of inappropriate antibiotic use. ARI diagnoses were stratified by patient age and trended over time. Five age categories were used: preschool children (0–4 years), school-aged children (5–17 years), young adults (18–24 years), adults (25–64 years) and older adults (65+ years). Visits with ARI diagnoses and total clinical encounters for each year under study are shown in Table 2. ARI counts from 2008 to 2011 are depicted in Figure 1.

**Table 2.** Acute respiratory infection (ARI) diagnoses as a percentage of all clinic visits, 2004–2011, by age group

Season	0–4 years			5–17 years			18–24 years			25–64 years			65+ years			Total		
	ARI	All visits	% ARI	ARI	All visits	% ARI	ARI	All visits	% ARI	ARI	All visits	% ARI	ARI	All visits	% ARI	ARI	All visits	% ARI
2004–05	15 243	48 781	31.2	17 512	81 024	21.6	6598	63 267	10.4	37 005	484 436	7.6	6180	129 911	4.8	82 538	807 419	10.2
2005–06	13 514	46 936	28.8	15 052	76 302	19.7	6016	60 962	9.9	32 066	484 920	6.6	5186	129 576	4.0	71 834	798 696	9.0
2006–07	13 604	47 656	28.5	15 566	78 563	19.8	5878	61 393	9.6	34 652	509 013	6.8	5222	137 139	3.8	74 922	833 764	9.0
2007–08	12 452	47 259	26.3	14 152	80 804	17.5	5680	62 810	9.0	36 010	532 544	6.8	5563	145 478	3.8	73 857	868 895	8.5
2008–09	11 148	44 094	25.3	12 856	81 800	15.7	4635	57 510	8.1	30 364	531 460	5.7	5244	148 184	3.5	64 247	863 048	7.4
2009–10	10 463	44 549	23.5	11 784	83 367	14.1	4341	55 231	7.9	29 317	547 346	5.4	5000	155 765	3.2	60 905	886 258	6.9
2010–11	9584	37 200	25.8	12 519	79 371	15.8	4317	51 430	8.4	31 740	539 787	5.9	5574	156 902	3.6	63 734	864 690	7.4
Total	86 008	316 475	27.2	99 441	561 231	17.7	37 465	412 603	9.1	231 154	3 629 506	6.4	37 969	1 002 955	3.8	492 037	5 922 770	8.3

All-cause respiratory infection diagnoses were used as a surrogate measure for ARI case counts in order to accurately capture respiratory pathogen activity. Because specific clinical and coded diagnoses in primary care settings are imprecise, erroneous or may reflect individual clinician prescribing behaviour [21], we felt that the all-cause metric more accurately reflected community trends in ARI activity.

### School attendance

Public primary, middle and high school calendars for all school districts served by clinics included in the CDW were examined to determine start and end dates for both the fall and spring school semesters. Partially due to Wisconsin state mandates, academic year start and end dates, as well as winter breaks were relatively uniform across the state with variation of less than 1 week between districts. School districts showed more variation in timing of spring vacations, so schools were assumed to remain in session throughout the spring semester for data analysis. Spring break typically lasts 9 days (5 school days), but varies widely over a 5-week period among school districts within any given year. As de-identified ARI events were aggregated over a wide geographic area, representing the catchment areas of 28 clinics and several dozen school districts, we were unable to assess for the differences in the aggregated data based on individual exposure to a particular school district break timing. School attendance for each week of every calendar year was coded with one of six values representing: (1) 1 week in session, (2) 2 weeks in session, (3) more than 2 consecutive weeks in session, (4) 1 week out of session, (5) 2 weeks out of session or (6) more than 2 consecutive weeks out of session.

### Meteorological data

Weekly average temperature and relative humidity data were obtained from the National Climatic Data Center for the period under study. As most clinics are located in the south-central portion of the state, meteorological data were obtained from the Dane County Regional Airport weather observation station (station ID 474 961, latitude 43°08′N, longitude 89°20′W).

### Statistical analysis

Data analysis was performed with R version 2.15.1 [22]. An over-dispersed Poisson generalised additive log-linear regression model was fit to the weekly number of ARI diagnoses as a function of school attendance as a categorical variable and temperature, relative humidity, year and season (calendar week within year) as smooth functions (thin plate regression splines) [23]. The number of ARI diagnoses in the prior week was also included as a covariate to account for possible autocorrelation. Analyses were conducted for all ages combined and for each age group individually. Risk ratios and associated 95% confidence intervals (CIs) were presented for each attendance category relative to the baseline category of more than 2 consecutive weeks out of school (i.e. summer vacation). A nominal *P*-value of 0.05 was regarded as statistically significant.

## Results

During the 7-year study period (2004–2011), 492 037 all-cause ARI cases were recorded, representing 8.3% from a total of 5 922 770 clinical visits. Of note was an inverse relationship between age and the predominance of ARI diagnoses; 27.2% of visits for children (ages 0–4) included an ARI diagnosis as compared with 3.8% of visits for adults age 65 years and older (see Table 2).

### School attendance

**Fig. 2.**
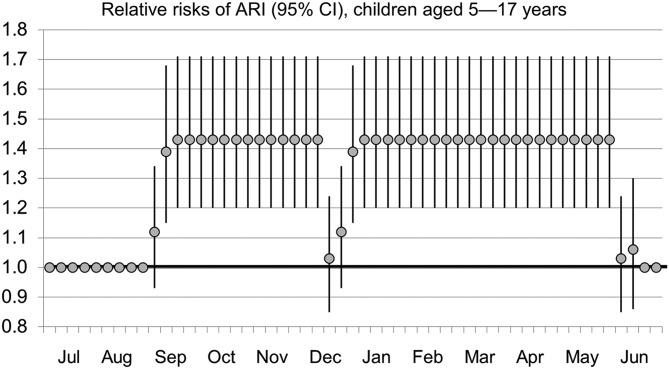
Relative risks of ARI (95% CI), children ages 5–17 years.

Risk ratios for ARI based on school attendance are presented in Table 3. Controlling for temperature and humidity, risks of ARI were increased for children 0–4 years and 5–17 years during school sessions. During the second week schools were in session, risk ratios were 1.12 (1.02–1.24) for children 0–4 years and 1.39 (1.15–1.68) for children 5–17 years. When schools were in session three or more consecutive weeks, risk ratios were 1.16 (1.06–1.27) and 1.43 (1.20–1.71) for children 0–4 years and 5–17 years, respectively. A similar finding was reflected in the pooled analysis of all ages, with risk ratios of 1.14 (1.04–1.24) for the first week schools were in session, 1.15 (1.05–1.26) for the second week and 1.15 (1.06–1.26) for more than 2 weeks into sessions. Risk ratios through a 12-month period for children ages 5–17 years are depicted in Figure 2.

**Table 3.** Relative risk of infection during in-school and out-of-school sessions, by age group

School session	Risk ratio (95% CI), by age category					
	0–4 years**	5–17 years**	18–24 years*	25–64 years*	>65 years	All ages**
Out for >2 weeks	1	1	1	1	1	1
In for 1 week	1.07 (0.97–1.18)	1.12 (0.93–1.34)	**1.18** (**1.06–1.31)**	**1.16** (**1.06–1.27)**	1.11 (0.98–1.26)	**1.14** (**1.04–1.24)**
In for 2 weeks	**1.12 (1.02–1.24)**	**1.39 (1.15–1.68)**	**1.13 (1.02–1.26)**	1.11 (1.00–1.22)	0.99 (0.86–1.13)	**1.15 (1.05–1.26)**
In for >2 weeks	**1.16 (1.06–1.27)**	**1.43 (1.20–1.71)**	1.07 (0.98–1.16)	1.09 (1.00–1.19)	0.98 (0.86–1.11)	**1.15 (1.06–1.26)**
Out for 1 week	1.07 (0.97–1.17)	1.03 (0.85–1.24)	1.05 (0.94–1.17)	1.02 (0.93–1.12)	0.92 (0.81–1.04)	1.03 (0.94–1.13)
Out for 2 weeks	**1.14 (1.03–1.27**)	1.06 (0.86–1.3)	1.03 (0.91–1.17)	1.05 (0.95–1.17)	0.98 (0.85–1.12)	1.07 (0.97–1.18)

Statistically significant (*P* < 0.05) risk ratios shown in bold.

*P-value < 0.05

**P-value < 0.01

For patients 18–24 years, risk ratios were 1.18 (1.06–1.31) and 1.13 (1.02–1.26) for the first and second weeks of school, respectively. For patients 25–64 years, the risk ratio for the first week of school was 1.16 (1.06–1.27).

There was no increase in risk ratio for out-of-school sessions for any age group with the exception of children 0–4 years, which showed a risk ratio of 1.14 (1.03–1.27) during the second week following the conclusion of the school year. Adults older than 65 did not show increased relative rates at any point during the year when adjusting for school attendance.

### Temperature and relative humidity

Once annual seasonality was modelled, neither temperature nor relative humidity was significantly associated with additional risk of infection (*P* > 0.05 in all age groups). These findings suggest that longer-term seasonal patterns are the predominant factors influencing disease activity and that these seasonal differences cannot be explained by short-term changes in temperature and/or relative humidity.

## Discussion

This study showed that the start of school sessions plays a significant role in seasonal outbreaks by increasing the risk of all-cause, medically attended ARIs. The effect is most pronounced in children 5–17 years, but is also seen in children 0–4 years and adults 18–64 years, which may suggest secondary household transmission. Comparisons among risks for differing age groups showed little difference for each of the time periods.

We used all-cause respiratory infection diagnoses as a proxy measure for ARI activity within a specific region to allow for an upper limit of respiratory pathogen activity. We included pneumonia and otitis media, as these diagnoses are often associated with viral aetiologies. For example, influenza vaccines reduce episodes of otitis media in children by 83% during the influenza season [24]. Utilising a common measure, however, prevents assessment of contributions from specific pathogens which may impart a stronger correlation with school attendance [25]. Other factors may have also influenced risk ratios. First, we used a wide-rage age category (25–64) based on the US Influenza Sentinel Providers Surveillance Network, which may have significantly affected the estimate of impact in younger adults, especially in parents of school-aged children. Second, we used a school calendar with mostly uniform annual autumn start dates and spring end dates. However, we could not account for spring school breaks, which may hamper transmission through school closure or conversely lead to higher risk due to increased travel [26]. Post-hoc graphical analysis demonstrated a consistent reduction in ARI visits for individuals age 5–17 years during the five potential weeks of spring break. Accordingly, our approach is conservative and likely underestimates school effect for the 5–17 year group. Parents may also be less likely to present to clinics during winter breaks either due to changes in care seeking behaviour or clinic closure.

Consolidating ARI aetiologies into a single category may also mask the contribution of temperature and humidity observed when studying specific pathogens. Enveloped viruses, for example, are known to be highly infectious at low relative humidity, whereas non-enveloped rhinoviruses have increased transmissibility at high relative humidity [14]. Because outbreaks of these viruses peak at different times and frequently overlap, underlying weather effects may be obscured. Further, the relationship between meteorological variables and virus infectivity may not be monotonic. Recent studies of influenza demonstrated a complex relationship to relative humidity [12, 27], and other meteorological variables such as absolute humidity can explain the seasonality of influenza in temperate climates [28]. Nevertheless, temperature and relative humidity are principal attributes of seasonality in temperate latitudes. Hence, the driving influences of these climatic factors were statistically accounted for by incorporating a seasonal term in the analysis. We were not able to isolate a significant role for short-term fluctuations in temperature and relative humidity.

Improved predictive models may allow forecasting of the anticipated volume of medically attended ARI visits, thus, allowing appropriate deployment of medical resources. Coupled with advanced community surveillance [29], public health mitigation efforts for significant outbreaks of respiratory infection may be more feasible. For example, if school exposures are shown to be an important cause of seasonal respiratory infectious disease outbreaks, then appropriately timed temporary school closures may be an effective way of disrupting local seasonal or pandemic outbreaks [30]. School closure appeared to be effective in preventing the spread of both severe acute respiratory syndrome [31] and H1N1 influenza [32–34]. Future research on seasonality and the contribution from school attendance will both help to establish a causative link and may improve predictive models for specific pathogens.

## References

[1] Lipsitch M and Viboud C (2009) Influenza seasonality: lifting the fog. Proceedings of the National Academy of Sciences of the United States of America 106, 3645–3646. 19276125 10.1073/pnas.0900933106 PMC2656132

[2] Grassly NC and Fraser C (2006) Seasonal infectious disease epidemiology. Proceedings of the Royal Society of London Series B, Biological Sciences 273, 2541–2550. 16959647 10.1098/rspb.2006.3604 PMC1634916

[3] Dingle JH (1953) A study of illness in a group of Cleveland families. I. Plan of study and certain general observations. American Journal of Hygiene 58, 16–30. 13065268 10.1093/oxfordjournals.aje.a119587

[4] van den Hof S, Conyn-van Spaendonck MA and van Steenbergen JE (2002) Measles epidemic in the Netherlands, 1999–2000. The Journal of Infectious Disease 186, 1483–1486. 10.1086/344894 12404165

[5] Fine PE and Clarkson JA (1982) Measles in England and Wales – I: an analysis of factors underlying seasonal patterns. International Journal of Epidemiology 11, 5–14. 7085179 10.1093/ije/11.1.5

[6] Chao DL, Halloran ME and Longini IM Jr (2010) School opening dates predict pandemic influenza A (H1N1) outbreaks in the United States. The Journal of Infectious Disease 202, 877–880. 10.1086/655810 PMC2939723 20704486

[7] Heymann AD (2009) School closure may be effective in reducing transmission of respiratory viruses in the community. Epidemiology and Infection 137, 1369–1376. 19351434 10.1017/S0950268809002556

[8] Monto AS (2004) Occurrence of respiratory virus: time, place and person. The Pediatric Infectious Disease Journal 23(suppl. 1), S58–S64. 14730271 10.1097/01.inf.0000108193.91607.34

[9] du Prel JB (2009) Are meteorological parameters associated with acute respiratory tract infections? Clinical Infectious Diseases: An Official Publication of the Infectious Diseases Society of America 49, 861–868. 19663691 10.1086/605435

[10] Yusuf S (2007) The relationship of meteorological conditions to the epidemic activity of respiratory syncytial virus. Epidemiology and Infection 135, 1077–1090. 17346359 10.1017/S095026880600776X PMC2870672

[11] Welliver RC Sr (2007) Temperature, humidity, and ultraviolet B radiation predict community respiratory syncytial virus activity. The Pediatric Infectious Disease Journal 26(suppl. 11), S29–S35. 18090197 10.1097/INF.0b013e318157da59

[12] Lowen AC (2007) Influenza virus transmission is dependent on relative humidity and temperature. PLoS Pathogens 3, 1470–1476. 17953482 10.1371/journal.ppat.0030151 PMC2034399

[13] Ji W (2011) Characteristics and the prevalence of respiratory viruses and the correlation with climatic factors of hospitalized children in Suzhou children's hospital. Zhonghua Yu Fang Yi Xue Za Zhi 45, 205–210. 21624230

[14] Karim YG (1985) Effect of relative humidity on the airborne survival of rhinovirus-14. Canadian Journal of Microbiology 31, 1058–1061. 3004682 10.1139/m85-199

[15] Davis GW (1971) Effect of relative humidity on dynamic aerosols of adenovirus 12. Applied Microbiology 21, 676–679. 4930277 10.1128/am.21.4.676-679.1971 PMC377254

[16] Casanova LM (2010) Effects of air temperature and relative humidity on coronavirus survival on surfaces. Applied and Environmental Microbiology 76, 2712–2717. 20228108 10.1128/AEM.02291-09 PMC2863430

[17] Weigl JA (2007) Ten years' experience with year-round active surveillance of up to 19 respiratory pathogens in children. European Journal of Pediatrics 166, 957–966. 17569085 10.1007/s00431-007-0496-x PMC7087302

[18] Bezerra PG (2011) Viral and atypical bacterial detection in acute respiratory infection in children under five years. PLoS One 6, e18928. 21533115 10.1371/journal.pone.0018928 PMC3078930

[19] Atmar RL (2012) Picornavirus, the most common respiratory virus causing infection among patients of all ages hospitalized with acute respiratory illness. Journal of Clinical Microbiology 50, 506–508. 22116142 10.1128/JCM.05999-11 PMC3264172

[20] Brittain-Long R (2011) Access to a polymerase chain reaction assay method targeting 13 respiratory viruses can reduce antibiotics: a randomised, controlled trial. BMC Medicine 9, 44. 21521505 10.1186/1741-7015-9-44 PMC3108322

[21] Vinson DC and Lutz LJ (1993) The effect of parental expectations on treatment of children with a cough: a report from ASPN. The Journal of Family Practice 37, 23–27. 8345335

[22] R Foundation for Statistical Computing (2012) R: A Language and Environment for Statistical Computing [Computer Program]. Vienna, Austria: R Foundation for Statistical Computing.

[23] Wood SN (2006) Generalized Additive Models : An introduction with R. Boca Raton, FL: Chapman & Hall/CRC.

[24] Heikkinen T (1991) Influenza vaccination in the prevention of acute otitis media in children. American Journal of Diseases of Children 145, 445–448. 1849344 10.1001/archpedi.1991.02160040103017

[25] McLean HQ (2017) School absenteeism among school-aged children with medically attended acute viral respiratory illness during three influenza seasons, 2012–2013 through 2014–2015. Influenza and Other Respiratory Viruses 11, 220–229. 27885805 10.1111/irv.12440 PMC5410714

[26] Polgreen PM (2010) A spatial analysis of the spread of mumps: the importance of college students and their spring-break-associated travel. Epidemiology and Infection 138, 434–441. 19737443 10.1017/S0950268809990719 PMC2956339

[27] Hanley BP and Borup B (2010) Aerosol influenza transmission risk contours: a study of humid tropics versus winter temperate zone. Virology Journal 7, 98. 20470403 10.1186/1743-422X-7-98 PMC2893155

[28] Shaman J (2010) Absolute humidity and the seasonal onset of influenza in the continental United States. PLoS Biology 8, e1000316. 20186267 10.1371/journal.pbio.1000316 PMC2826374

[29] Fowlkes A (2013) Estimating influenza incidence and rates of influenza-like illness in the outpatient setting. Influenza and Other Respiratory Viruses 7, 694–700. 22984820 10.1111/irv.12014 PMC5781202

[30] Community Preventive Services Task Force (2017) Emergency Preparedness and Response: School Dismissals to Reduce Transmission of Pandemic Influenza. Available at https://www.thecommunityguide.org/findings/emergency-preparedness-and-response-school-dismissals-reduce-transmission-pandemic-influenza (Accessed 22 February 2017).

[31] Chan KP (2005) Control of severe acute respiratory syndrome in Singapore. Environmental Health and Preventive Medicine 10, 255–259. 21432128 10.1007/BF02897699 PMC2723408

[32] Calatayud L (2010) Pandemic (H1N1) 2009 virus outbreak in a school in London, April-May 2009: an observational study. Epidemiology and Infection 138, 183–191. 19925691 10.1017/S0950268809991191

[33] Earn DJ (2012) Effects of school closure on incidence of pandemic influenza in Alberta, Canada. Annals of Internal Medicine 156, 173–181. 22312137 10.7326/0003-4819-156-3-201202070-00005

[34] Copeland DL (2013) Effectiveness of a school district closure for pandemic influenza A (H1N1) on acute respiratory illnesses in the community: a natural experiment. Clinical Infectious Diseases: An Official Publication of the Infectious Diseases Society of America 56, 509–516. 23087391 10.1093/cid/cis890

